# Role of immune-checkpoint inhibitors in lung cancer

**DOI:** 10.1177/1753465817750075

**Published:** 2018-01-31

**Authors:** Prantesh Jain, Chhavi Jain, Vamsidhar Velcheti

**Affiliations:** Cleveland Clinic, Cleveland, OH, USA; Cleveland Clinic, Cleveland, OH, USA; Cleveland Clinic, 9500 Euclid Avenue, Cleveland, OH 44195, USA

**Keywords:** biomarkers, checkpoints, immunotherapy, lung cancer

## Abstract

Immune checkpoint inhibitors, mainly drugs targeting the programmed cell death 1 (PD-1)/programmed cell death ligand 1 (PD-L1) and cytotoxic T-lymphocyte antigen 4 (CTLA4) pathways, represent a remarkable advance in lung cancer treatment. Immune checkpoint inhibitors targeting PD-1 and PD-L1 are approved for the treatment of patients with non-small-cell lung cancer, with impressive clinical activity and durable responses in some patients. This review will summarize the mechanism of action of these drugs, the clinical development of these agents and the current role of these agents in the management of patients with lung cancer. In addition, the review will discuss the role of predictive biomarkers for optimal patient selection for immunotherapy and management of autoimmune side effects of these agents.

## Introduction

Cancer immunotherapy has its origins in the early 1900s when the concepts of host immune defense against cancer and cancer immune surveillance were postulated.^1,2^ Since the early 1900s there were attempts at using techniques to stimulate the immune system against cancer using intratumoral injections of live or an inactivated mixture of *Streptococcus pyogenes* and *Serratia marcecsens*.^1^ Despite some early success with such approaches, lack of understanding of the mechanisms of the antitumor immune response and the complexity involved in such an approach limited the enthusiasm. It took nearly a century to unravel the mysteries of the immune system and the role of the immune system in cancer.^3^ In response to an inflammatory signal (secondary to infection or cancer), the antigen-presenting cells (APCs) responding to the antigen are stimulated by the proinflammatory cytokines [interleukin (IL)-1, tumor necrosis factor (TNF)-α] and the APCs interact with naïve T cells. This interaction between APCs or tumor cells and T cells allows for the proliferation of the antigen-specific T cells and is the critical first step in mounting an immune response. Following this interaction between APCs or tumor cells with cells in the adaptive immune system (T cells, B cells) resulting in subsequent immune response *via* a complex orchestration of immune coregulatory pathways. These intricate coregulatory pathways are often redundant mechanisms to avoid immune response against self antigens. These coregulatory pathways, namely immune checkpoints, are coopted by the tumor cells to avoid the immune system.^4–8^ Recent advances in our understanding of these key immune regulatory pathways resulted in the development of promising new strategies in treating cancer.

Lung cancer is the world’s leading cause of cancer death.^9^ Platinum-doublet chemotherapy has been the standard of care for frontline therapy in advanced non-small cell lung cancer (NSCLC) without oncogenic drivers. Five-year survival for these patients is dismal at under 10%. In about 15–20% of patients with NSCLC key genomic alterations leading to oncogenic activation, which is amenable to targeted therapy, can be identified. However, most of these patients receiving targeted drugs will have an emergence of resistance to targeted therapy.^10,11^ Recently, understanding the host immune system–tumor interactions has led to the acknowledgment of immune evasion as an additional hallmark of cancer.^12^ Several immune cell types within the tumor microenvironment serve complex and paradoxical roles from the antitumor response, influence tumorigenesis and immune evasion. But the key immune regulatory pathways, which serve as the critical immune evasion interface between the tumor and the immune cells, are promising targets for drug development.^8^ The recent success of drugs targeting the immune-checkpoint pathways, particularly the programmed cell death 1 (PD-1) pathway, has changed the paradigm of clinical management of several cancers.^8^ Treatment with immunotherapy has the potential to induce clinically meaningful and durable responses.^13–16^ Three drugs targeting the PD-1 pathway (nivolumab, pembrolizumab, and atezolizumab) have been approved by the US Food and Drug Administration (FDA) for use in both chemotherapy-naïve and previously treated advanced stage NSCLC.^17–20^ A timeline of FDA approval for checkpoint inhibitors (CPIs) in lung cancer is presented in Table 1. Immune checkpoint blockade with PD-1/programmed cell death ligand 1 (PD-L1) inhibitors has thus become part of the standard-of-care treatment option for patients with advanced stage NSCLC; however, only a small subset (20–30%) of patients respond to treatment.^16–25^

**Table 1.** Timeline for FDA approval of checkpoint inhibitors.

Drug	Manufacturer	FDA approval	Indication	Companion diagnostic
Nivolumab	Bristol-Myers Squibb (Princeton, New Jersey)	March 2015	Second-line advanced stage NSCLC (squamous cell carcinoma)	None required
Nivolumab	Bristol-Myers Squibb	October 2015	Second-line advanced stage NSCLC (nonsquamous cell carcinoma)	None required
Pembrolizumab	Merck (Kenilworth, New Jersey)	October 2015	Second-line advanced stage NSCLC	PD-L1 IHC >1% TPS*
Atezolizumab	Genentech/Roche (San Francisco, California)	April 2016	Second-line advanced stage NSCLC	None required
Pembrolizumab	Merck	October 2016	First-line advanced stage NSCLC	PD-L1 IHC >50% TPS
Pembrolizumab with carboplatin/pemetrexed	Merck	May 2017	First-line advanced stage NSCLC (nonsquamous cell carcinoma)	None required

FDA, US Food and Drug Administration; IHC, immunohistochemistry; NSCLC, non-small cell lung cancer; PD-1, programmed cell death 1; PD-L1 programmed cell death ligand 1; TPS, tumor proportion score.

## Immune checkpoint pathways

**Figure 1.**
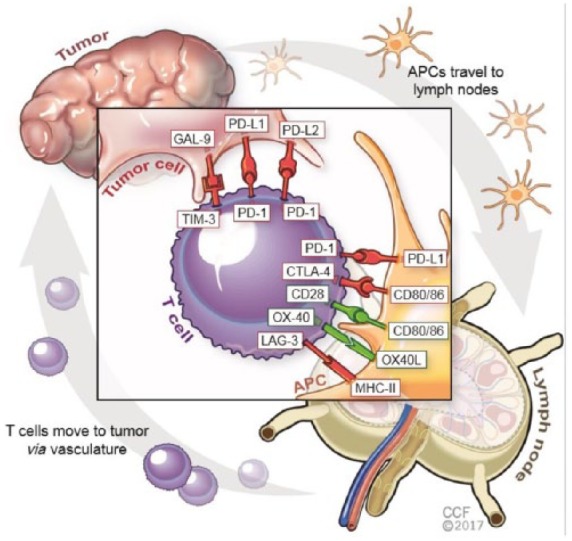
Pathways involved in immune checkpoint regulation. APC, antigen-presenting cell; PD-1, programmed cell death 1 [co-stimulatory signals (green)]; PD-L1, programmed cell death ligand 1 [co-inhibitory signals (red)].

Cancer immunotherapy is based on improved tumor antigen presentation and recognition; stimulation or amplification of an immune response; or disinhibition of immune cells to allow for an improved antitumor immune response.^8^ Immune response begins with antigen presentation by APCs such as dendritic cells that present tumor antigens on the cell surface with major histocompatibility complex (MHC) molecules. APCs present antigens to T cells by MHC peptide complexes to antigen-specific T-cell receptors on the surface. Various regulatory mechanisms check the proliferation of autoreactive T cells and maintenance of immune tolerance in normal tissues. This intricate balance between immune-stimulatory and inhibitory signals limit harmful autoimmune responses.^26^ Tumors utilize multiple mechanisms of immune evasion, such as genetic and epigenetic modifications; expression of immune inhibitory cytokines such as IL-10 and transforming growth factor β in the tumor microenvironment; and induction of T-cell suppressive signaling pathways.^8^ The inhibitory signals to suppress T-cell activity are mediated by ‘immune-checkpoint’ molecules (inhibitory ligands and their cognate receptors), including the CD28/cytotoxic T-lymphocyte antigen 4 (CTLA-4) axis, and PD-L1/PD-1 which have emerged as promising druggable targets (Figure 1). Other checkpoint molecules such as TIM3, B7H3, VISTA, LAG3, and TIGIT are currently being evaluated as potential targets for cancer immunotherapy.

1. PD-1/PD-L1 pathway: PD-1 is a coinhibitory surface receptor that is expressed by activated and exhausted T cells. It is also expressed on other immune cells such as B lymphocytes, natural killer (NK) cells, and myeloid derived suppressor cells (MDSCs).^27,28^ Interaction between PD-1 and its ligands, PD-L1 and PD-L2, on tumor cells leads to downregulation of T-cell response in the tumor microenvironment^29,30^ (Figure 1). Many lung cancer cells overexpress PD-L1 as a mechanism for suppressing T-cell response.^7,29^
2. CD28/CTLA-4 system of immune modulation: CTLA-4 is expressed mainly on T cells (CD4+, helper and CD8+, killer T cells) with some expression in other immune cells including B lymphocytes and fibroblasts.^31,32^ CTLA-4 competes with the costimulatory receptor CD28 for binding to the same ligands, B7-1 (CD80) and B7-2 (CD86) on the surface of APCs, resulting in downregulation of immune response^32,33^ (Figure 1). CTLA-4 acts early during the priming phase of antigen presentation and following T-cell receptor–peptide complex engagement, it is rapidly mobilized to the cell surface, allowing feedback inhibition to occur within an hour of antigen presentation.^34^ Therapeutic anti-CTLA-4 monoclonal antibodies have shown clinical activity in advanced melanoma, most likely *via* disrupting the CD28 activation on T cells as well as through depletion of regulatory T cells (T-regs) in the tumor microenvironment.^35^

## PD-1 blocking antibodies

Anti-PD-1 antibodies block the interaction of PD-1 with PD-L1 and PDL-2, but do not prevent PD-L1 interaction with CD80 (B7.1).

## Nivolumab

Nivolumab (BMS-936558) is a fully human immunoglobulin G4 (IgG4) antibody against PD-1. In an early phase I trial (Checkmate-003 study), nivolumab demonstrated promising clinical efficacy, particularly in patients with high PD-L1 expression.^25,36,37^ Results from two landmark studies, CheckMate-017 (squamous NSCLC) and CheckMate-057 (nonsquamous NSCLC), demonstrated benefit in progression-free survival (PFS) and overall survival (OS) from nivolumab compared with docetaxel.^17,18^ Checkmate-017 is a randomized phase III clinical trial in patients with squamous cell lung carcinoma evaluating nivolumab *versus* docetaxel in patients previously treated with a platinum-doublet chemotherapy. In this study, nivolumab demonstrated a 1-year survival rate of 42% [95% confidence interval (CI) 34–50] compared with 24% (95% CI 17–31) in the docetaxel group. The OS was significantly longer with nivolumab, with a 41% reduction in the risk of death with nivolumab [hazard ratio (HR) 0.59; 95% CI 0.44–0.79; *p* < 0.001]. In addition, overall response rate (ORR) was higher in the nivolumab arm compared with docetaxel [20% (95% CI 14–28) *versus* 9% (95% CI 5–15); *p* = 0.008].^18^ Checkmate-057 is a randomized phase III clinical trial in patients with nonsquamous cell lung carcinoma evaluating nivolumab *versus* docetaxel in patients previously treated with platinum-doublet chemotherapy. In this study, nivolumab demonstrated a 1-year survival rate of 51% (95% CI 45–56%) compared with 39% (95% CI 33–45%) in the docetaxel group. The OS was significantly longer with nivolumab, with a 27% reduction in the risk of death with nivolumab (HR 0.73; 96% CI 0.59–0.89; *p* = 0.002). In addition, ORR was higher in the nivolumab arm compared with docetaxel [19% (95% CI 15–24) *versus* 12% (95% CI 9–17), *p* = 0.02].

In both the CheckMate-017 and CheckMate-057 trials the predictive role of PD-L1 expression was evaluated in the following subgroups: at least 1%, at least 5%, or at least 10% tumor cell expression using Dako 28-8 assay. In the CheckMate-017 trial, PD-L1 expression at any level was not predictive of clinical benefit. However, in the CheckMate-057 trial, the was a trend to improve efficacy in patients with higher expression of PD-L1. There was no statistically significant difference demonstrated in OS in patients lacking PD-L1 expression. Dako 28.8 PD-L1 assay is approved as a complementary diagnostic test for patients with nonsquamous NSCLC but not for squamous NSCLC.

These positive trial results led to approval of nivolumab by the FDA in advanced squamous and nonsquamous NSCLC as second-line systemic therapy after progression on first-line chemotherapy, regardless of PD-L1 expression (Table 1).

However, a phase III trial (CheckMate-26) comparing nivolumab with platinum-based doublet chemotherapy as first-line treatment for stage IV, recurrent NSCLC with at least 1% PD-L1 positive, showed no benefit in the primary endpoint, PFS [median PFS 4.2 (CI 3.0–5.6) *versus* 5.9 (CI 5.4–6.9) months; HR 1.15 (CI 0.91–1.45); *p* = 0.2511]. The ORR for nivolumab was 26.1% *versus* 33.5% for chemotherapy. Nivolumab did not improve PFS or OS in a retrospective subgroup analysis done on patients with at least 5% PD-L1 positivity.^38^

## Pembrolizumab

Pembrolizumab (MK-3475) is a fully humanized IgG4 monoclonal antibody against PD-1. In a phase Ib trial (Keynote-001 study), pembrolizumab demonstrated promising clinical efficacy, particularly in patients with high PD-L1 expression.^13^ A subsequent open-label randomized phase II/III clinical trial (Keynote-10) compared pembrolizumab with docetaxel in patients with metastatic NSCLC whose disease had progressed on prior chemotherapy and expressed PD-L1 staining of at least 1% tumor proportion score. In this study, the median OS and PFS were both significantly improved with pembrolizumab compared with docetaxel [OS: HR 0.71 (95% CI 0.58–0.88, *p* = 0.0008) with 2 mg/kg; HR 0.61 (95% CI 0.49–0.75, *p* < 0.0001) with 10 mg/kg] and the benefit was even higher for the subgroup of patients with PD-L1 of at least 50% tumor proportion score (TPS) [OS: HR 0.54 (95% CI 0.38–0.77, *p* = 0.0002) in patients receiving 2 mg/kg every 3 weeks; HR 0.50 (95% CI 0.36–0.70, *p* < 0.0001) with 10 mg/kg of pembrolizumab every 3 weeks].^19^ In the frontline setting, pembrolizumab monotherapy was compared with platinum-doublet chemotherapy in a phase-randomized open-label clinical trial (Keynote-024) in patients with advanced stage NSCLC with PD-L1 expression (⩾50% TPS).^39^ In this trial patients were randomized to either pembrolizumab 200 mg every 3 weeks or investigator’s choice of platinum-doublet chemotherapy, with patients in the control arm allowed to cross over to receive pembrolizumab. The study met its primary endpoint of PFS, demonstrating improvement in PFS with pembrolizumab (10.3 months, 95% CI 6.7–not reached) compared with platinum-doublet chemotherapy (6.0 months, 4.2–6.2). PFS was longer in the pembrolizumab-treated patients *versus* patients treated with chemotherapy (HR for disease progression 0.50; 95% CI 0.37–0.68; *p* < 0.001). In addition, 80.2% (95% CI 72.9–85.7) of patients in the pembrolizumab arm were alive at 6 months compared with 72.4% (95% CI 64.5–78.9) in the chemotherapy arm. OS was significantly improved in patients treated with pembrolizumab, with decreased risk of death compared with chemotherapy (HR 0.60; 95% CI 0.41–0.89; *p* = 0.005).^39^ In a phase II clinical trial (Keynote-21) in patients with chemotherapy-naïve advanced nonsquamous NSCLC, carboplatin-pemetrexed with continued pemetrexed maintenance was compared with the same regimen combined with pembrolizumab.^40^

Based on these clinical trial data, pembrolizumab is approved by the FDA in advanced squamous and nonsquamous NSCLC as first-line systemic therapy for patients with PD-L1 expression [22C3 immunohistochemical staining (IHC) with >50% TPS) or as a second-line systemic therapy after progression on first-line chemotherapy, with at least 1% PD-L1 expression on tumor cells. Pembrolizumab was recently also FDA approved in the first-line setting for metastatic nonsquamous NSCLC in combination with pemetrexed and carboplatin independent of PD-L1 expression (Table 1).

## PD-L1 blocking antibodies

Anti-PD-L1 antibodies block the interaction of PD-L1 with PD-1 and CD80 (B7.1), but do not prevent the interaction of PD-L2 with PD-1 and CD80 with CTLA-4.

## Atezolizumab

Atezolizumab (MPDL-3280A) is a humanized IgG1 monoclonal antagonistic antibody that targets PD-L1. It is engineered to bypass antibody-dependent cell-mediated cytotoxicity (ADCC) of activated T cells that express PD-L1. In a phase I trial with expansion cohorts in patients with NSCLC, atezolizumab demonstrated promising clinical efficacy.^41^ In an open-label phase II randomized clinical trial (POPLAR), patients with advanced stage NSCLC whose disease progressed on post-platinum chemotherapy were assigned to receive either atezolizumab or docetaxel once every 3 weeks.^42^ In this study, patients treated with atezolizumab had an improved median OS of 12.6 months (95% CI 9.7–16.4) *versus* 9.7 months (8.6–12.0) for docetaxel [HR 0.73 (95% CI 0.53–0.99); *p* = 0.04].^42^ A confirmatory phase III (OAK) trial in patients with advanced stage NSCLC following progression on platinum-based chemotherapy compared atezolizumab with docetaxel. Like the POPLAR results, the OS was significantly improved with atezolizumab in comparison to docetaxel [median OS 13.8 months (95% CI 11.8–15.7) *versus* 9.6 months (8.6–11.2); HR 0.73 (95% CI 0.62–0.87), *p* = 0.0003]. PD-L1 expression on tumor cells (TC1/2/3) or immune cells (IC1/2/3) (⩾1% PD-L1 by VENTANA SP142 assay) was predictive of the benefit of atezolizumab. In patients with TC1/2/3 or IC1/2/3 PD-L1 expression in advanced NSCLC, atezolizumab improved OS with a median OS of 15.7 months (95% CI 12.6–18.0) *versus* 10.3 months (8.8–12.0) with docetaxel [HR 0.74 (95% CI 0.58–0.93); *p* = 0.0102]. However, patients lacking PD-L1 expression (TC0 and IC0) also had improved survival with atezolizumab [OS 12.6 months *versus* 8.9 months; HR 0.75 (95% CI 0.59–0.96)].^20^

These results led to approval of atezolizumab by the FDA in the second-line setting for patients with advanced stage NSCLC and VENTANA SP142 assay was approved as a complimentary diagnostic (Table 1).

## Durvalumab

Durvalumab (MEDI4736) is a high-affinity, humanized IgG1κ antagonistic antibody that targets PD-L1. Results from a phase II study (ATLANTIC) showed preferential activity in tumors with PD-L1 expression.^43^ PD-L1 positivity was defined as at least 25% of tumor cells with membranous staining for PD-L1. Response rate was 16.4% in patients with PD-L1-positive tumors and 7.5% in PD-L1-negative tumors that received durvalumab. The randomized phase III, PACIFIC trial [ClinicalTrials.gov identifier: NCT02125461] of durvalumab as sequential treatment in patients with locally advanced, unresectable NSCLC whose disease did not progress following definitive platinum-based concurrent chemoradiation showed median PFS improvement of over 11 months from time of randomization [16.8 months *versus* 5.6 months; HR 0.52 (95% CI 0.42–0.65); *p* < 0.001], regardless of PD-L1 expression.^44^ The OS data are still pending. In another phase III trial (MYSTIC) of frontline therapy in patients with NSCLC, durvalumab monotherapy is being compared with either a combination of durvalumab plus the anti-CTLA-4 antibody tremelimumab or standard-of-care chemotherapy [ClinicalTrials.gov identifier: NCT02453282]. The results of this trial are still awaited. Combination therapies of durvalumab with other agents, including gefitinib, AZD9291 and other immunotherapies (Table 3), are also being evaluated in phase I trials.

## PD-1 and CTLA-4 blocking antibodies

### Ipilimumab and tremelimumab

Ipilimumab is an IgG1 CTLA-4 monoclonal antibody from Bristol-Myers Squibb (Princeton, New Jersey) that did not show efficacy in patients with NSCLC. It is currently being investigated in multiple combination trials discussed in a subsequent section of this review. Another humanized monoclonal antibody targeting CTLA-4 is tremelimumab (AstraZeneca, Cambridge, UK). In patients with advanced or metastatic NSCLC, tremelimumab was compared with best supportive care in maintenance setting [ClinicalTrials.gov identifier: NCT00312975], with no difference in PFS.^45^ Tremelimumab is currently being evaluated in combination with durvalumab and other immunotherapeutic agents discussed in further detail in a subsequent section.

Thus far, data from frontline trials using CPIs in lung cancer would justify prescription of pembrolizumab to patients with at least 50% tumor cells positive for PD-L1 staining; and chemotherapy for those who are do not show this level of staining. The value of other assays for selection of frontline patients including exploratory analyses is unproven.

## Combined immune-checkpoint inhibition

Due to distinct mechanisms of activation of immune checkpoints on T cells, such as CTLA-4 stimulation in the lymphatic tissue whereas PD-1/PD-L1 activation occurs in the tumor microenvironment,^8^ there is rationale for combining CPIs for improved clinical outcomes. Hellman and colleagues described the results of a phase I study combining nivolumab plus ipilimumab as first-line treatment for advanced NSCLC, with patients assigned to either nivolumab (3 mg/kg) every 2 weeks plus ipilimumab (1 mg/kg) every 12 weeks or nivolumab (3 mg/kg) every 2 weeks plus ipilimumab (1 mg/kg) every 6 weeks (CheckMate 012).^46^ The response rate of 57% was achieved in both arms, with at least 1% PD-L1 expression compared with 47% and 38% in the total population. No significant difference was observed in median PFS, with values of 8.1 (5.6–13.6) and 3.9 (2.6–13.2) months, respectively. No previously known toxicities were reported and grade 3–4 adverse events occurred in 37% and 33% in both arms. A phase III clinical trial to evaluate this combination is currently ongoing (CheckMate 227) [ClinicalTrials.gov identifier: NCT02477826].

A combination of durvalumab (anti-PD-L1 antibody) and tremelimumab (anti-CTLA-4 antibody) is being evaluated for safety, tolerability and antitumor activity in a phase Ib trial with 102 patients [ClinicalTrials.gov identifier: NCT02000947].^47^ Clinical benefit was observed regardless of PD-L1 expression status and objective responses were achieved in 23% of patients in the tremelimumab 1 mg/kg cohort. Durvalumab 20 mg/kg every 4 weeks plus tremelimumab 1 mg/kg was defined as the maximum tolerated dose, and selected for ongoing phase III studies (MYSTIC, NEPTUNE) [ClinicalTrials.gov identifier: NCT02453282, NCT02542293].^48^

## Combination with chemotherapy

Chemotherapy can modulate immune responses directly or indirectly by immunostimulation, increased immunogenicity through increased mutational burden and neo-epitope formation.^49^ Immune CPIs work by reactivating immune responses. The mechanism of action of chemotherapeutic drugs and CPIs may therefore be complementary.

CheckMate 012 and CheckMate 227 are phase I and phase III trials, respectively, evaluating a combination of nivolumab plus chemotherapy in the frontline setting.^50,51^ Keynote-021, a phase II study, reported that the addition of pembrolizumab to carboplatin/pemetrexed in newly diagnosed metastatic nonsquamous NSCLC increased ORR to 55% (42–68%) compared with 29% (18–41%), regardless of PD-L1 status. Grade 3–4 adverse events did not differ significantly by addition of pembrolizumab.^40^ This led to the FDA approval of pembrolizumab in combination with carboplatin/pemetrexed in patients with newly diagnosed stage IV nonsquamous NSCLC. This combination is being further evaluated in phase III trials (Keynote-189, Keynote-407) [ClinicalTrials.gov identifier: NCT02578680, NCT02775435]. A combination of ipilimumab (in a phased regimen) with carboplatin and paclitaxel in first-line therapy of advanced or metastatic NSCLC improved PFS (5.1 *versus* 4.2 months) and ORR (32% *versus* 14%) [ClinicalTrials.gov identifier: NCT01285609]. At present, conclusions from chemotherapy combinations with CPIs are limited by small numbers of patients and limited follow-up times. Although response rate and PFS are increased when pembrolizumab is added to chemotherapy, OS remains unchanged.

## Role of biomarkers in patient selection

Cancer immunotherapy has changed conventional treatment paradigms by expanding the treatment options for patients with cancer. However, despite current success, the response rate to CPIs in advanced NSCLC is around 30%. Thus, there is a growing need to identify predictive and prognostic biomarkers for better patient selection. The basic principles underlying a good biomarker include analytical validity (reliability and reproducibility), as well as clinical utility. Several studies in NSCLC and melanoma show that tumor response to CPIs is associated with their immune profiles. For example, tumors that have high T-cell infiltration and express an inflammatory gene signature and show a ‘T-cell inflamed phenotype’ are more amenable to checkpoint inhibition.^52^

## PD-L1 expression

PD-L1 is the ligand for checkpoint receptor PD-1 expressed on T cells. Tumors with high infiltration of T cells may demonstrate higher PD-L1 expression as a form of adaptive resistance mechanism and are more likely to benefit from PD-1/PD-L1 inhibition.^7,25,53–57^ At least 50% PD-L1 expression is approved as a companion biomarker (Dako 22C3 pharmDx) with frontline, single-agent pembrolizumab in patients with NSCLC.^13,39^ Other complementary diagnostic tests (recommended, but not required for drug prescription) for nivolumab (Dako 28-8 pharmDx) and atezolizumab (Ventana SP142) are also FDA approved for use in NSCLC.^58^ Despite approval of IHC assays and evaluation of PD-L1 expression on tumor cells or immune cells to predict the efficacy of PD-1/PD-L1 blockade,^59–61^ its clinical utility as an exclusive predictive biomarker remains controversial. While most studies concur that a higher level of tumor cell membrane PD-L1 expression is associated with improved outcome/response to PD-1/PD-L1 blockade, there is evidence that a subpopulation of patients with PD-L1-negative tumors may also have clinical benefit from CPIs.^60^ The heterogeneity and dynamics of PD-L1 expression confound its use as a predictive biomarker. Different IHC staining assays utilize different antibody clones and scoring systems for PD-L1 detection, as summarized in Table 2. The Ventana SP263, Dako 22C3 and Dako 28-8 clones have been used most commonly and were found to cluster together when evaluated for the pathologists’ concordance in scoring using NSCLC specimens.^61,62^ The SP142 clone did not cluster with the others and seemed to underscore PD-L1 expression compared with the other assays.^61,62^ One explanation is that this clone was raised against an intracellular epitope of PD-L1, whereas the others target extracellular epitopes of PD-L1.

**Table 2.** Diagnostic assays for PD-L1 for anti-PD-1/PD-L1 drugs in non-small cell lung cancer.

	Pembrolizumab			Nivolumab	Atezolizumab
Assay	22C3			28–8	SP142
Indication	1st	1st	2nd	2nd	2nd
PD-L1 required	⩾50%	No	⩾1%	No^$^	No^$^
Regimen	Single agent	With chemo*	Single agent	Single agent	Single agent

* With carboplatin and pemetrexed for adenocarcinomas only.

$ Response is enriched when positive.

PD-1, programmed cell death 1; PD-L1 programmed cell death ligand 1.

**Table 3.** Immune-related adverse events associated with checkpoint inhibition and management.

Manifestation	Severity	Management
**Gastrointestinal (GI)** • Immune-mediated colitis • Pancreatitis	• **Grade 1:** Asymptomatic imaging finding of colon thickening • **Grade 2:** Moderate abdominal symptoms 4–6 stools/day	• Hold immunotherapy for grade ⩾2; work up to rule out infectious etiology ova, parasites and stool culture. Stool antigen for *Clostridium difficile* • American Diet Association colitis diet, loperamide or atropine sulfate • If persistent symptoms over 1 week start oral prednisone 1 mg/kg/day or equivalent. Taper over 4 weeks if symptoms improve. Start infliximab 5 mg/kg every 2 weeks if symptoms do not improve after 3 days on steroid treatment
	• **Grade 3/4:** Severe and persistent abdominal pain, fever, ileus and life-threatening complications >7 stools/day over baseline	• Strongly recommend GI consult and colonoscopy to rule out nonimmune etiologies • Recommend hospitalization and start intravenous methyl prednisone 2–4 mg/kg/day or equivalent, taper over 4–6 weeks if resolves to grade 1 or better. If no improvement after 48–72 h, add alternative immunosuppressive agents mycophenolate mofetil or infliximab
**Liver** • Immune-mediated hepatitis	**Grade 1:** Asymptomatic/mildly symptomatic AST/ALT <2.5 × ULN Total bilirubin <1.5 × ULN **Grade 2:** Symptomatic AST/ALT: 2.5–5 × ULN Total bilirubin: 1.5–3 × ULN	• Delay drug; increase frequency of LFT monitoring until resolution • Oral prednisone 1 mg/kg/day or equivalent, taper over 4 weeks if symptoms resolve, add alternative immunosuppressive agent (tacrolimus, cyclophosphamide or mycophenolate mofetil) if symptoms do not improve after 48 h. Avoid infliximab because of potential for hepatotoxicity. • Consider cautious restarting of immunotherapy after LFTs improve to grade 1 or lower
	**Grade 3/4:** Symptomatic AST/ALT >5 × ULN Total bilirubin >3 × ULN	• Recommend hospitalization and start intravenous methyl prednisone 2–4 mg/kg/day or equivalent, taper over 4–6 weeks if resolves to grade 1 or better. If no improvement after 48–72 h, add alternative immunosuppressive agents tacrolimus, cyclophosphamide or mycophenolate mofetil. Avoid infliximab due to potential for hepatotoxicity
***Endocrine*** • Thyroiditis • Hypothyroidism • Hyperthyroidism • Hypophysitis • Hypopituitarism • Adrenal insufficiency	• **Grade 1:** Asymptomatic or mild symptoms; clinical or laboratory finding only • **Grade 2**: Moderate; limiting age-appropriate instrumental ADL	**Thyroiditis:** treat hypothyroidism and hyperthyroidism per standard guidelines: does not require holding immunotherapy **Adrenal insufficiency:** physiologic replacement doses of steroids; however, if presenting in adrenal crisis/shock, admit to the hospital and start stress dose steroids, and intravenous fluids. Rule out sepsis. Immunotherapy may be resumed when stable and on physiologic doses of adrenal replacement **Hypophysitis:** prednisone 1–2 mg/kg/day or equivalent with a slow taper; consultation with endocrine recommended; patients may require hormone replacement therapy for life
	• **Grade 3/4:** Severe or medically significant and life-threatening (grade 4); disabling and limiting ADL and self-care	
**Skin** • Dermatitis	**Grade 1:** <10% BSA; asymptomatic **Grade 2**: 10–30% BSA Mildly symptomatic	• Administer topical steroids and oral antihistaminic drugs • If unresolved with above measures consider low dose systemic corticosteroids and consider treatment break if no improvement; consider dermatology consultation
	**Grade 3/4:** >30% BSA Severe: Stevens-Johnson syndrome, necrolysis, or rash with dermal ulcerations or necrotic, hemorrhagic manifestations	• Discontinue drug; administer systemic corticosteroid therapy of 1–2 mg/kg/day of prednisone or equivalent • Dermatology consultation • Hold immunotherapy until resolved to grade 1
**Pulmonary** • Immune-mediated pneumonitis	• **Grade 1:** Asymptomatic imaging finding only • **Grade 2:** Moderate symptoms with limited interference with activities of day to day living	• Hold immunotherapy for 3–4 weeks; if asymptomatic monitor for symptoms closely. • If new symptoms develop, oral prednisone 1 mg/kg/day or equivalent and taper over 4–6 weeks after symptoms improve
	• **Grade 3/4:** Severe symptoms limiting activities of day to day living; hypoxia and respiratory failure	• Recommend hospitalization and pulmonary consultation and start intravenous methyl prednisone 2–4 mg/kg/day or equivalent, taper over 4–6 weeks if resolves to grade 1 or better. If no improvement after 48–72 h, consider bronchoscopy with BAL/transbronchial biopsy to rule out other etiology; if negative add alternative immunosuppressive agents mycophenolate mofetil or infliximab
**Renal** • Autoimmune nephritis	• **Grade 1:** Asymptomatic, increase in creatinine above the baseline but ⩽1.5 ULN • **Grade 2:** Increase in creatinine above 1.5 ULN ⩽3	• Continue immunotherapy for grade 1; closely monitor renal function and electrolyte imbalances. Rule out other etiology for renal failure. • Hold immunotherapy for grade 2 and above; start 0.5–1 mg/kg/day of prednisone or equivalent
	• **Grade 3:** Increase in creatinine above 3 ULN ⩽6 • **Grade 4:** Increase in creatinine above 6 ULN	• Consult nephrology; renal biopsy. • Hold immunotherapy permanently; start 1.0–2.0 mg/kg/day of prednisone or equivalent

ADL, activities of daily living; ALT, alanine transaminase; AST, aspartate transaminase; BAL, bronchoalveolar lavage; BSA, body surface area; LFT, liver function test; ULN, upper limit of normal.

Another caveat with the use of PD-L1 expression as a biomarker is variability in PD-L1 expression (both inter- and intratumor heterogeneity) at different sites of disease, such as primary *versus* metastatic sites, and different time points during the treatment course (i.e. before or after chemotherapy). The use of fresh *versus* archival biopsies may also affect PD-L1 expression.^63–66^ Differences in PD-L1 expression were detected between biopsied specimens and surgically resected tumors from the same patient.^67^ Different biopsies coming from the same lung in patients with multifocal lung cancer showed discordant expression of PD-L1 in about one third of the total patient population.^54^ Nonetheless, PD-L1 expression remains an important factor in achieving response to PD-1 blockade. In NSCLC, patients with high levels of PD-L1 tumor staining achieved an excellent response to PD-1 blockade.

## Mutational and neoantigen load

Lung cancer is predicted to be a highly immunogenic tumor, expressing many neoantigens, and is responsive to checkpoint inhibition.^68^ Rizvi and colleagues showed that higher mutational burden was associated with stable response lasting over 6 months in patients with NSCLC receiving pembrolizumab.^69^ Over 178 nonsynonymous mutations and neoantigen burden were associated with prolonged OS. Several studies later demonstrated that in addition to the high mutational burden, low neoantigen intratumoral heterogeneity might also be an important factor. McGranahan and colleagues analyzed The Cancer Genome Atlas database on NSCLC adenocarcinoma and showed that a combination of high mutational burden and low neoantigen intratumoral heterogeneity (<1%) is more significantly associated with longer survival time (irrespective of treatment).^70^ High mutation burden is an increasingly important emerging biomarker for identification of patients for checkpoint immunotherapy. Although promising, prospective studies are warranted to confirm these approaches and other investigational biomarkers for patient selection in routine clinical use. PD-L1 status alone is not sufficient to rule in or rule out the use of CPIs and further investigation to combine two or more methods to capture the immune status might be more efficient as a composite predictive biomarker for immune CPI therapy.

## Spectrum of immune-related toxicities and management

Immune-checkpoint pathways play a critical physiologic role in maintaining self tolerance and preventing autoimmunity. Immune CPIs thus have the potential to alter the immune homeostasis and result in autoimmune side effects, termed as immune-related adverse events (irAEs). These side effects can encompass a wide range of manifestations, which can affect almost all tissues and organs. IrAEs mostly affect the joints (arthritis), colon (colitis), lung (pneumonitis), endocrine glands (endocrinopathies), skin (dermatitis), and liver (hepatitis). In general, most toxicities associated with PD-1 and PD-L1 agents are easily managed with a high dose of corticosteroids and are rarely refractory to immunosuppressive treatments. However, in some patients, particularly when not detected early, irAEs can be life threatening.^71^

Assessing the severity of irAEs is critical for effective management of these unusual toxicities that often require a multidisciplinary approach. Toxicities should be graded using the Common Terminology Criteria for Adverse Events developed by the National Cancer Institute (Table 3). Most irAEs are mild and asymptomatic (grade 1) and patients can continue treatments in most situations with close monitoring without immunosuppression. However, this is dependent on the specific organ involvement, for example in patients with grade 1 pneumonitis (asymptomatic radiographic findings only), treatment should be stopped, and patients should be monitored closely with a repeat computed tomography scan of the chest within 3 weeks to confirm resolution prior to restarting treatment. Patients with grade 2 irAEs typically require their treatment to be stopped temporarily and they should be monitored closely after initiation of oral prednisone of 0.5–1.0 mg/kg/day or the equivalent as an outpatient. Patients should be evaluated frequently for any worsening symptoms. Patients will need to be tapered off steroids very slowly over 4–6 weeks. Prophylaxis for *Pneumocystis jirovecii* pneumonia should be considered in these patients. Patients with grade 3/4 irAEs have significant risk of mortality and morbidity and hence hospitalization should be considered for initial management; these patients often require permanent discontinuation of treatment. These patients require 1–2 mg/kg dose of methylprednisolone or the equivalent for initial management until toxicity resolves to grade 2 or lower; they may require additional nonsteroidal immunosuppressive agents like mycophenolate mofetil or TNF inhibitors (infliximab) if no response is seen with the high doses of steroids within 72 h. Depending on the clinical situation, additional system-focused diagnostic studies like colonoscopies, liver biopsies, bronchoscopy with bronchoalveolar lavage and transbronchial biopsies may be necessary to rule out other etiologies. Patients with irAEs may often require multidisciplinary care. Effective management of irAEs requires heightened awareness of these toxicities among not just the oncologist but also nononcology specialists who are involved in the care of patients treated with immunotherapy.

In summary, recent approval of immune CPIs in the management of advanced stage NSCLC is a significant advancement in treatment of NSCLC. In addition, the exciting results from immunotherapy strategies has opened exciting possibilities for future immunotherapy combination strategies that will possibly yield even more effective treatment strategies and keep our hope alive to achieve a cure for at least a subset of patients with advanced NSCLC.

## References

[1] Ribatti D. The concept of immune surveillance against tumors. The first theories. Oncotarget 2017; 8: 7175–7180. 27764780 10.18632/oncotarget.12739 PMC5351698

[2] Ehrlich P. Ueber den jetzigen Stand der Karzinomforschung. Ned Tijdschr Geneeskd 1909; 5: 273–290.

[3] D’Errico G Machado HL Sainz B. A current perspective on cancer immune therapy: step-by-step approach to constructing the magic bullet. Clin Transl Med 2017; 6: 3. 28050779 10.1186/s40169-016-0130-5 PMC5209322

[4] Schalper KA Velcheti V Carvajal D In situ tumor PD-L1 mRNA expression is associated with increased TILs and better outcome in breast carcinomas. Clin Cancer Res 2014; 20: 2773–2782. 24647569 10.1158/1078-0432.CCR-13-2702

[5] Schalper KA Brown J Carvajal-Hausdorf D Objective measurement and clinical significance of TILs in non-small cell lung cancer. J Natl Cancer Inst 2015; 107: 1–4. 10.1093/jnci/dju435 PMC4565530 25650315

[6] Velcheti V Rimm DL Schalper KA. Sarcomatoid lung carcinomas show high levels of programmed death ligand-1 (PD-L1). J Thorac Oncol 2013; 8: 803–805. 23676558 10.1097/JTO.0b013e318292be18 PMC3703468

[7] Velcheti V Schalper KA Carvajal DE Programmed death ligand-1 expression in non-small cell lung cancer. Lab Invest 2014; 94: 107–116. 24217091 10.1038/labinvest.2013.130 PMC6125250

[8] Velcheti V Schalper K. Basic overview of current immunotherapy approaches in cancer. Am Soc Clin Oncol Educ Book 2016; 35: 298–308. 27249709 10.1200/EDBK_156572

[9] Torre LA Siegel RL Jemal A. Lung cancer statistics. Adv Exp Med Biol 2016; 893: 1–19. 26667336 10.1007/978-3-319-24223-1_1

[10] Lovly CM Iyengar P Gainor JF. Managing resistance to EFGR- and ALK-targeted therapies. Am Soc Clin Oncol Educ Book 2017; 37: 607–618. 28561721 10.1200/EDBK_176251 PMC10183098

[11] Jain P Khanal R Sharma A Afatinib and lung cancer. Exp Rev Anticancer Ther 2014; 14: 1391–1406. 10.1586/14737140.2014.983083 25417728

[12] Hanahan D Weinberg RA. Hallmarks of cancer: the next generation. Cell 2011; 144: 646–674. 21376230 10.1016/j.cell.2011.02.013

[13] Garon EB Rizvi NA Hui R Pembrolizumab for the treatment of non-small-cell lung cancer. N Engl J Med 2015; 372: 2018–2028. 25891174 10.1056/NEJMoa1501824

[14] Lipson EJ Forde PM Hammers HJ Antagonists of PD-1 and PD-L1 in cancer treatment. Sem Oncol 2015; 42: 587–600. 10.1053/j.seminoncol.2015.05.013 PMC4555873 26320063

[15] Motzer RJ Escudier B McDermott DF Nivolumab versus everolimus in advanced renal-cell carcinoma. N Engl J Med 2015; 373: 1803–1813. 26406148 10.1056/NEJMoa1510665 PMC5719487

[16] Topalian SL Sznol M McDermott DF Survival, durable tumor remission, and long-term safety in patients with advanced melanoma receiving nivolumab. J Clin Oncol 2014; 32: 1020–1030. 24590637 10.1200/JCO.2013.53.0105 PMC4811023

[17] Borghaei H Paz-Ares L Horn L Nivolumab versus docetaxel in advanced nonsquamous non-small-cell lung cancer. N Engl J Med 2015; 373: 1627–1639. 26412456 10.1056/NEJMoa1507643 PMC5705936

[18] Brahmer J Reckamp KL Baas P Nivolumab versus docetaxel in advanced squamous-cell non-small-cell lung cancer. N Engl J Med 2015; 373: 123–135. 26028407 10.1056/NEJMoa1504627 PMC4681400

[19] Herbst RS Baas P Kim DW Pembrolizumab versus docetaxel for previously treated, PD-L1-positive, advanced non-small-cell lung cancer (KEYNOTE-010): a randomised controlled trial. Lancet (London, England) 2016; 387: 1540–1550. 10.1016/S0140-6736(15)01281-7 26712084

[20] Rittmeyer A Barlesi F Waterkamp D Atezolizumab versus docetaxel in patients with previously treated non-small-cell lung cancer (OAK): a phase 3, open-label, multicentre randomised controlled trial. Lancet (London, England) 2017; 389: 255–265. 10.1016/S0140-6736(16)32517-X PMC6886121 27979383

[21] Hamid O Robert C Daud A Safety and tumor responses with lambrolizumab (anti-PD-1) in melanoma. N Engl J Med 2013; 369: 134–144. 23724846 10.1056/NEJMoa1305133 PMC4126516

[22] Hodi FS O’Day SJ McDermott DF Improved survival with ipilimumab in patients with metastatic melanoma. N Engl J Med 2010; 363: 711–723. 20525992 10.1056/NEJMoa1003466 PMC3549297

[23] Lynch TJ Bondarenko I Luft A Ipilimumab in combination with paclitaxel and carboplatin as first-line treatment in stage IIIB/IV non-small-cell lung cancer: results from a randomized, double-blind, multicenter phase II study. J Clin Oncol 2012; 30: 2046–2054. 22547592 10.1200/JCO.2011.38.4032

[24] Postow MA Chesney J Pavlick AC Nivolumab and ipilimumab versus ipilimumab in untreated melanoma. N Engl J Med 2015; 372: 2006–2017. 25891304 10.1056/NEJMoa1414428 PMC5744258

[25] Topalian SL Hodi FS Brahmer JR Safety, activity, and immune correlates of anti-PD-1 antibody in cancer. N Engl J Med 2012; 366: 2443–2454. 22658127 10.1056/NEJMoa1200690 PMC3544539

[26] Igney FH Behrens CK Krammer PH. Tumor counterattack: concept and reality. Eur J Immunol 2000; 30: 725–731. 10741386 10.1002/1521-4141(200003)30:3<725::AID-IMMU725>3.0.CO;2-D

[27] Keir ME Butte MJ Freeman GJ PD-1 and its ligands in tolerance and immunity. Annu Rev Immunol 2008; 26: 677–704. 18173375 10.1146/annurev.immunol.26.021607.090331 PMC10637733

[28] Schalper KA Carvajal-Hausdorf D McLaughlin J Clinical significance of PD-L1 protein expression on tumor-associated macrophages in lung cancer. J ImmunoTher Cancer 2015; 3: P415.

[29] Pardoll DM. The blockade of immune checkpoints in cancer immunotherapy. Nat Rev Cancer 2012; 12: 252–264. 22437870 10.1038/nrc3239 PMC4856023

[30] Sznol M Chen L. Antagonist antibodies to PD-1 and B7-H1 (PD-L1) in the treatment of advanced human cancer: response. Clin Cancer Res 2013; 19: 5542. 24048329 10.1158/1078-0432.CCR-13-2234 PMC6101650

[31] Allison JP Krummel MF. The Yin and Yang of T cell costimulation. Science (New York, NY) 1995; 270: 932–933. 10.1126/science.270.5238.932 7481795

[32] Quandt D Hoff H Rudolph M A new role of CTLA-4 on B cells in thymus-dependent immune responses in vivo. J Immunol 2007; 179: 7316–7324. 18025174 10.4049/jimmunol.179.11.7316

[33] Schreiber RD Old LJ Smyth MJ. Cancer immunoediting: integrating immunity’s roles in cancer suppression and promotion. Science (New York, NY) 2011; 331: 1565–1570. 10.1126/science.1203486 21436444

[34] Topalian SL Drake CG Pardoll DM. Immune checkpoint blockade: a common denominator approach to cancer therapy. Cancer Cell 2015; 27: 450–461. 25858804 10.1016/j.ccell.2015.03.001 PMC4400238

[35] Selby MJ Engelhardt JJ Quigley M Anti-CTLA-4 antibodies of IgG2a isotype enhance antitumor activity through reduction of intratumoral regulatory T cells. Cancer Immunol Res 2013; 1: 32–42. 24777248 10.1158/2326-6066.CIR-13-0013

[36] Gettinger SN Horn L Gandhi L Overall survival and long-term safety of nivolumab (anti-programmed death 1 antibody, BMS-936558, ONO-4538) in patients with previously treated advanced non-small-cell lung cancer. J Clin Oncol 2015; 33: 2004–2012. 25897158 10.1200/JCO.2014.58.3708 PMC4672027

[37] Kazandjian D Suzman DL Blumenthal G FDA approval summary: nivolumab for the treatment of metastatic non-small cell lung cancer with progression on or after platinum-based chemotherapy. Oncologist 2016; 21: 634–642. 26984449 10.1634/theoncologist.2015-0507 PMC4861371

[38] Carbone DP Reck M Paz-Ares L First-line nivolumab in stage IV or recurrent non-small-cell lung cancer. N Engl J Med 2017; 376: 2415–2426. 28636851 10.1056/NEJMoa1613493 PMC6487310

[39] Reck M Rodriguez-Abreu D Robinson AG Pembrolizumab versus chemotherapy for PD-L1-positive non-small-cell lung cancer. N Engl J Med 2016; 375: 1823–1833. 27718847 10.1056/NEJMoa1606774

[40] Langer CJ Gadgeel SM Borghaei H Carboplatin and pemetrexed with or without pembrolizumab for advanced, non-squamous non-small-cell lung cancer: a randomised, phase 2 cohort of the open-label KEYNOTE-021 study. Lancet Oncol 2016; 17: 1497–1508. 27745820 10.1016/S1470-2045(16)30498-3 PMC6886237

[41] Herbst RS Soria JC Kowanetz M Predictive correlates of response to the anti-PD-L1 antibody MPDL3280A in cancer patients. Nature 2014; 515: 563–567. 25428504 10.1038/nature14011 PMC4836193

[42] Fehrenbacher L Spira A Ballinger M Atezolizumab versus docetaxel for patients with previously treated non-small-cell lung cancer (POPLAR): a multicentre, open-label, phase 2 randomised controlled trial. Lancet (London, England) 2016; 387: 1837–1846. 10.1016/S0140-6736(16)00587-0 26970723

[43] Garassino M Vansteenkiste J Kim JH Durvalumab in ⩾3rd-line locally advanced or metastatic, EGFR/ALK wild-type NSCLC: results from the phase 2 ATLANTIC study. J Thorac Oncol 2017; 12: S10–S11.

[44] Antonia SJ Villegas A Daniel D Durvalumab after chemoradiotherapy in stage III non-small-cell lung cancer. N Engl J Med 2017; 377: 1919–1929. 28885881 10.1056/NEJMoa1709937

[45] Zatloukal P Heo DS Park K Randomized phase II clinical trial comparing tremelimumab (CP-675,206) with best supportive care (BSC) following first-line platinum-based therapy in patients (pts) with advanced non-small cell lung cancer (NSCLC). J Clin Oncol 2009; 27: 8071.

[46] Hellmann MD Rizvi NA Goldman JW Nivolumab plus ipilimumab as first-line treatment for advanced non-small-cell lung cancer (CheckMate 012): results of an open-label, phase 1, multicohort study. Lancet Oncol 2017; 18: 31–41. 27932067 10.1016/S1470-2045(16)30624-6 PMC5476941

[47] Antonia S Goldberg SB Balmanoukian A Safety and antitumour activity of durvalumab plus tremelimumab in non-small cell lung cancer: a multicentre, phase 1b study. Lancet Oncol 2016; 17: 299–308. 26858122 10.1016/S1470-2045(15)00544-6 PMC5500167

[48] Mok T Schmid P De Castro G P2. 06–022 first-line durvalumab plus tremelimumab vs platinum-based chemotherapy for advanced/metastatic NSCLC: phase 3 NEPTUNE study. J Thorac Oncol 2017; 12: S1084.

[49] Galluzzi L Zitvogel L Kroemer G. Immunological mechanisms underneath the efficacy of cancer therapy. Cancer Immunol Res 2016; 4: 895–902. 27803050 10.1158/2326-6066.CIR-16-0197

[50] Paz-Ares L Brahmer J Hellmann M 144TiPCheckMate 227: a randomized, open-label phase 3 trial of nivolumab, nivolumab plus ipilimumab, or nivolumab plus chemotherapy versus chemotherapy in chemotherapy-naïve patients with advanced non-small cell lung cancer (NSCLC). Ann Oncol 2017; 28(1), mdx091.064.

[51] Rizvi NA Hellmann MD Brahmer JR Nivolumab in combination with platinum-based doublet chemotherapy for first-line treatment of advanced non-small-cell lung cancer. J Clin Oncol 2016; 34: 2969–2979. 27354481 10.1200/JCO.2016.66.9861 PMC5569693

[52] Weber JS. Biomarkers for checkpoint inhibition. Am Soc Clin Oncol Educ Book 2017; 37: 205–209. 28561695 10.1200/EDBK_175463

[53] Gibney GT Weiner LM Atkins MB. Predictive biomarkers for checkpoint inhibitor-based immunotherapy. Lancet Oncol 2016; 17: e542–e551. 27924752 10.1016/S1470-2045(16)30406-5 PMC5702534

[54] Mansfield AS Murphy SJ Peikert T Heterogeneity of programmed cell death ligand 1 expression in multifocal lung cancer. Clin Cancer Res 2016; 22: 2177–2182. 26667490 10.1158/1078-0432.CCR-15-2246 PMC4854782

[55] Taube JM Klein A Brahmer JR Association of PD-1, PD-1 ligands, and other features of the tumor immune microenvironment with response to anti-PD-1 therapy. Clin Cancer Res 2014; 20: 5064–5074. 24714771 10.1158/1078-0432.CCR-13-3271 PMC4185001

[56] Teng MW Ngiow SF Ribas A Classifying cancers based on T-cell infiltration and PD-L1. Cancer Res 2015; 75: 2139–2145. 25977340 10.1158/0008-5472.CAN-15-0255 PMC4452411

[57] Schalper KA Carvajal-Hausdorf D McLaughlin J Differential expression and significance of PD-L1, IDO-1, and B7-H4 in human lung cancer. Clin Cancer Res 2017; 23: 370–378. 27440266 10.1158/1078-0432.CCR-16-0150 PMC6350535

[58] Grigg C Rizvi NA. PD-L1 biomarker testing for non-small cell lung cancer: truth or fiction? J Immunother Cancer 2016; 4: 48. 27532023 10.1186/s40425-016-0153-x PMC4986262

[59] Danilova L Wang H Sunshine J Association of PD-1/PD-L axis expression with cytolytic activity, mutational load, and prognosis in melanoma and other solid tumors. Proc Natl Acad Sci USA 2016; 113: E7769–E7777. 27837027 10.1073/pnas.1607836113 PMC5137776

[60] Daud AI Loo K Pauli ML Tumor immune profiling predicts response to anti-PD-1 therapy in human melanoma. J Clin Invest 2016; 126: 3447–3452. 27525433 10.1172/JCI87324 PMC5004965

[61] Ratcliffe MJ Sharpe A Midha A Agreement between programmed cell death ligand-1 diagnostic assays across multiple protein expression cutoffs in non-small cell lung cancer. Clin Cancer Res 2017; 23: 3585–3591. 28073845 10.1158/1078-0432.CCR-16-2375

[62] Hirsch FR McElhinny A Stanforth D PD-L1 immunohistochemistry assays for lung cancer: results from phase 1 of the blueprint PD-L1 IHC assay comparison project. J Thorac Oncol 2017; 12: 208–222. 27913228 10.1016/j.jtho.2016.11.2228

[63] McLaughlin J Han G Schalper KA Quantitative assessment of the heterogeneity of PD-L1 expression in non-small-cell lung cancer. JAMA Oncol 2016; 2: 46–54. 26562159 10.1001/jamaoncol.2015.3638 PMC4941982

[64] Patel SP Kurzrock R. PD-L1 expression as a predictive biomarker in cancer immunotherapy. Mol Cancer Ther 2015; 14: 847–856. 25695955 10.1158/1535-7163.MCT-14-0983

[65] Sheng J Fang W Yu J Expression of programmed death ligand-1 on tumor cells varies pre and post chemotherapy in non-small cell lung cancer. Sci Rep 2016; 6: 20090. 26822379 10.1038/srep20090 PMC4731819

[66] Twyman-Saint Victor C Rech AJ Maity A Radiation and dual checkpoint blockade activate non-redundant immune mechanisms in cancer. Nature 2015; 520: 373–377. 25754329 10.1038/nature14292 PMC4401634

[67] Ilie M Long-Mira E Bence C Comparative study of the PD-L1 status between surgically resected specimens and matched biopsies of NSCLC patients reveal major discordances: a potential issue for anti-PD-L1 therapeutic strategies. Ann Oncol 2016; 27: 147–153. 26483045 10.1093/annonc/mdv489

[68] Schumacher TN Schreiber RD. Neoantigens in cancer immunotherapy. Science (New York, NY) 2015; 348: 69–74. 10.1126/science.aaa4971 25838375

[69] Rizvi NA Hellmann MD Snyder A Cancer immunology: mutational landscape determines sensitivity to PD-1 blockade in non-small cell lung cancer. Science (New York, NY) 2015; 348: 124–128. 10.1126/science.aaa1348 PMC4993154 25765070

[70] McGranahan N Furness AJ Rosenthal R Clonal neoantigens elicit T cell immunoreactivity and sensitivity to immune checkpoint blockade. Science (New York, NY) 2016; 351: 1463–1469. 10.1126/science.aaf1490 PMC4984254 26940869

[71] Michot JM Bigenwald C Champiat S Immune-related adverse events with immune checkpoint blockade: a comprehensive review. Eur J Cancer (Oxford, England: 1990) 2016; 54: 139–148. 10.1016/j.ejca.2015.11.016 26765102

